# *OsLAC12*, a Laccase-Encoding Gene, Contributes to Rice Heat Tolerance Through Regulation of Antioxidant Defense and Secondary Metabolism

**DOI:** 10.3390/plants14182846

**Published:** 2025-09-12

**Authors:** Shuangcheng Ding, Zihui Gao, Yuhang Chen, Jiaxin Liu, Yuxin Xue, Yulu Teng, Simin Qin, Xiaohai Tian, Hongwei Wang

**Affiliations:** 1 MARA Key Laboratory of Sustainable Crop Production in the Middle Reaches of the Yangtze River (Co-Construction by Ministry and Province), Yangtze University, Jingzhou 434025, China; shchding@yangtzeu.edu.cn; 2 Agricultural College, Yangtze University, Jingzhou 434025, China; 18091235588@163.com (Z.G.); chenyuhang820@163.com (Y.C.); 18535927844@163.com (J.L.); 15872743050@163.com (Y.X.); 19846968186@163.com (Y.T.); 19071388367@163.com (S.Q.)

**Keywords:** laccase activity, heat stress, lignin biosynthesis, ROS, thermotolerance

## Abstract

Heat stress (HT) exerts significant negative impacts on plant growth, development, and productivity. In this study, we identified *OsLAC12*, a heat-induced laccase-encoding gene, as a key regulator of heat tolerance in rice. Functional validation confirmed that *OsLAC12* encodes an active laccase, with supporting evidence showing that roots of *OsLAC12* overexpression lines exhibited ~1.5-fold higher laccase activity and nearly three times the lignin content compared to the wild type (WT). Under HT, *oslac12* mutants showed significantly lower seedling survival rates (~13–22%) than WT (~43%), whereas OE lines displayed higher survival rates (~68–74%). At the heading stage, *OsLAC12* overexpression lines activated the antioxidant enzyme system in spikelets, regulated reactive oxygen species (ROS) levels in anthers, and mitigated HT-induced damage to pollen viability. In contrast, *oslac12* mutants showed an average 10.8% reduction in pollen viability compared to WT, accompanied by significantly lower seed-setting rates. Collectively, our findings demonstrate that the laccase *OsLAC12* positively regulates rice resistance to HT stress, providing critical insights for improving plant thermotolerance through genetic improvement of cell wall-associated pathways.

## 1. Introduction

Rice (*Oryza sativa* L.), a stable cereal crop worldwide, is increasingly threatened by declining productivity due to the growing frequency and intensity of high-temperature events, which are further exacerbated by climate change [1]. During the vegetative growth stage, elevated temperatures disrupt critical physiological processes such as photosynthesis and respiration. This disruption manifests as visible symptoms including leaf yellowing and wilting, followed by reduced chlorophyll content and, in severe cases, plant death [2]. Studies have demonstrated that for every 1 °C increase in the minimum nighttime temperature during the reproductive stage, rice yield decreases by 10% [3]. Additionally, when temperatures exceed 35 °C for more than 2 h on the day of flowering, spikelet fertility is significantly reduced, thereby severely impacting the seed-setting rate [4,5]. The HT-induced decline in spikelet fertility is primarily attributed to impaired pollination [6]. Notably, HT damages the normal cellular structure and physiological functions of the tapetum, hinders anther maturation and dehiscence, and inhibits both pollen germination on the stigma and pollen tube elongation [7,8]. Thus, gaining insights into the molecular mechanisms underlying heat tolerance and identifying candidate genes associated with heat tolerance are essential for enhancing rice heat resistance.

In response to HT, plants have evolved a diverse array of molecular mechanisms involving signal transduction and the expression of stress-resistance genes. Heat-induced reactive oxygen species (ROS) bursts act as early signaling molecules, enabling cells to mount rapid responses [9,10]. However, excessive ROS accumulation can lead to irreversible cellular oxidative damage, including membrane lipid peroxidation, DNA damage, chloroplast degradation, and ultimately, cell death [1,11]. It has been reported that programmed cell death (PCD) is primarily regulated by ROS levels in anthers [12]. Consequently, ROS accumulation in anthers has emerged as a key physiological indicator of reduced pollen viability and impaired fertility in rice exposed to high-temperature stress during meiosis. HT-induced ROS accumulation is primarily counteracted by a robust antioxidant defense system, which comprises various enzymatic and non-enzymatic antioxidants [13]. Among these, enzymatic antioxidants—including peroxidase (POD), catalase (CAT), superoxide dismutase (SOD), and ascorbate peroxidase (APX)—are generally recognized as the most effective [14]. The distinction between increased and decreased activity of these enzymes directly reflects stress adaptation: elevated SOD/POD/APX activity enhances ROS scavenging to protect cells, while reduced activity indicates impaired antioxidant capacity and heightened oxidative damage [14]. Many studies indicated that the enhancement of antioxidant enzyme activity is one of the reasons for the improvement of heat tolerance in plants. Thus, maintaining low cellular ROS levels is critical for plants carrying stress-resistance genes to sustain their tolerance under high-temperature conditions.

Recent studies have highlighted dynamic changes in plant cell walls as critical players in sensing and responding to HT [15,16]. Lignin, a vital component of plant secondary cell walls, contributes to stem structural integrity and enhances resistance to both biotic stresses (e.g., diseases) and abiotic stresses [15]. Lignin is synthesized through the polymerization of monolignols, primarily p-coumaryl, coniferyl, and sinapyl alcohols, via the phenylpropanoid pathway [17]. These monolignols are derived from the general phenylpropanoid pathway, which typically starts with the aromatic amino acid phenylalanine produced by the shikimate pathway [17]. Like lignin, flavonoid metabolism is the second branch of the phenylpropanoid pathways. However, a complex regulatory relationship exists within this pathway, where shifts in metabolic flux toward lignin synthesis and/or flavonoid accumulation depending on enzyme activity and stress signals. Thus, exploring the intricate regulation mechanism of phenylpropanoids can enhance plants developmental cues and stress responses.

Laccase (EC 1.10.3.2, LAC), a copper-dependent oxidoreductase, serves as a key enzyme in catalyzing the oxidation of monolignols and facilitating their polymerization into lignin polymers [18,19]. Plant laccases form a large family of oxidases characterized by three conserved blue copper protein domains [20]. To date, laccase gene family members have been identified in various species: for example, 17 *LAC* genes in *Arabidopsis thaliana*, 30 in rice, 27 in *Sorghum bicolor*, and 45 in *Triticum aestivum* [21,22,23,24]. Mounting evidence links laccases to lignin polymerization and decomposition. In Arabidopsis, silencing of *LAC17* and *LAC14* significantly reduced lignin biosynthesis and resulted in an irregular xylem phenotype [25]. Additionally, simultaneous knockout of *LAC11*, *LAC4*, and *LAC17* in Arabidopsis severely impaired plant growth, leading to narrowed root diameters, closed anthers, stunted vascular tissue development, and a marked reduction in lignification [26]. Laccases have also been implicated in seed coat lignification in Cleome species [27,28]. Beyond their role in lignification, numerous laccase genes are induced by abiotic stresses, including extreme temperatures (low/high), drought, and exposure to hormones, as well as during defense responses [21,29,30]. For instance, *ZmLAC3* and *ZmLAC5* in maize significantly increase lignin content and lodging resistance under high-nitrogen conditions [31]. Overexpression of rice laccase gene *OsChI1* enhances salt and drought tolerance in transgenic Arabidopsis [32], while ectopic expression of another rice laccase, *OsLAC10*, improves copper stress tolerance in Arabidopsis [22]. Recent studies also demonstrate that *CsLAC18* in citrus positively regulates cold stress responses [33]. Similarly, Cai et al. reported that *laccase2* in Arabidopsis is involved in responses to abiotic stresses such as drought [21], and Xu et al. showed that citrus laccase genes are induced by abiotic stresses (low/high temperature, drought) and hormone treatments (ABA, MeJA, SA) [34]. Despite extensive evidence linking laccases to plant abiotic stress tolerance, their involvement in high-temperature stress responses and the underlying mechanisms remain poorly understood. Carbohydrates supply carbon skeletons for lignin synthesis and cell wall remodeling, thereby playing crucial roles in plant responses to HT. However, the potential links between laccases and carbohydrate transport/utilization under HT remain unclear. Additionally, studies exploring how HT-induced metabolic flux redirection affects distinct branches of the phenylpropanoid pathway, as well as its relationship with perturbations in other secondary metabolic processes, remain inconclusive. To address this gap, we systematically investigated the function of the rice laccase gene *OsLAC12* (LOC_Os03g18640), as previously annotated [22]. Our findings reveal that *OsLAC12* plays a key role in enhancing rice heat tolerance: its overexpression regulates the accumulation of lignin and flavonoids, which contribute to improved thermotolerance. These results provide valuable insights for breeding programs aimed at developing heat-tolerant rice varieties, offering a novel target to enhance yield stability amid increasingly frequent and severe high-temperature events.

## 2. Results

### 2.1. OsLAC12 Is SA, ABA, and Heat Stress Inducibale

**Figure 1:**
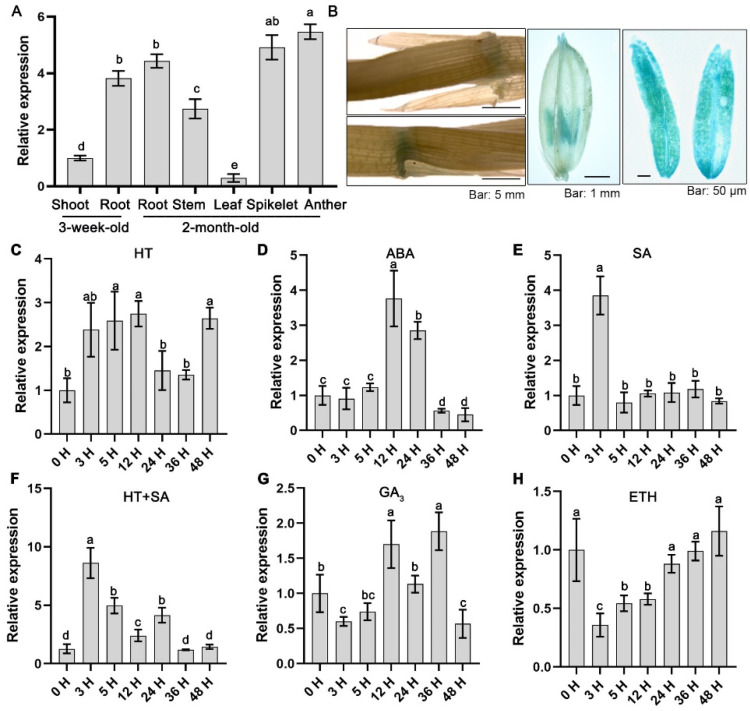
Expression patterns of *OsLAC12*. (**A**) qRT-PCR analysis of *OsLAC12* in different tissues during different developmental stages. (**B**) GUS staining of *proOsLAC12:GUS* transgenic rice T_3_ plants. (**C**) Expression of *OsLAC12* in response to 45 °C heat stress. (**D**) Expression of *OsLAC12* in response to 100 μM ABA. (**E**) Expression of *OsLAC12* in response to 10 mM SA. (**F**) Expression of *OsLAC12* under 45 °C heat stress, with simultaneous salicylic acid soaking treatment. (**G**) Expression of *OsLAC12* in response to 10 mM GA_3_. (**H**) Expression of *OsLAC12* in response to 10 mM ETH (ethylene). Rice Tubulins gene transcript level was used as an internal control for data normalization. Data were presented as mean ± SD, based on three independent biological replicates. Bars with different letters indicate statistically significant differences (*p* ≤ 0.05, ANOVA with Duncan’s test).

To clarify the spatiotemporal expression pattern of *OsLAC12*, we first analyzed its relative expression levels in three-week-old wild-type Nipponbare rice. Quantitative real-time PCR (qRT-PCR) analysis revealed that *OsLAC12* was highly expressed in underground tissues at the seedling stage, with expression levels 3.8-fold higher than those in aboveground tissues (Figure 1A). At the heading stage, *OsLAC12* showed the highest expression in panicles, followed by roots and stems, with the lowest expression in leaves. Next, to further characterize the tissue-specific expression pattern of *OsLAC12*, a 1.5 kb upstream DNA fragment of *OsLAC12* was fused with the β-glucuronidase (GUS) reporter gene, and the construct was introduced into rice. Histochemical GUS staining was performed on positive T_3_ transgenic lines. As shown in Figure 1B, *OsLAC12* was strongly expressed in the leaf pulvini and anthers of rice. Finally, three-week-old rice seedlings were subjected to various abiotic stresses and hormone treatments to investigate *OsLAC12* expression dynamics. When *Tubulin* was used as an internal control, results showed that *OsLAC12* expression was induced by HT, with a ~2-fold increase within 12 h (Figure 1C). Additionally, *OsLAC12* expression was significantly induced by ABA and SA, reaching a maximum ~4-fold increase relative to controls (Figure 1D,E). Importantly, *OsLAC12* showed a robust response to HT after SA pretreatment, with an ~8.0-fold increase at 3 h compared to unstressed plants (Figure 1F). In contrast, *OsLAC12* exhibited only mild responsiveness to gibberellin (GA) and ethephon (Figure 1G,H). As shown in Figure S1, when *18S rRNA* was used as an internal control, the *OsLAC12* expression patterns normalized by these two internal controls were largely consistent. Collectively, these observations suggest that *OsLAC12* may play a role in plant responses to abiotic stresses, particularly in SA-, ABA-, and heat-mediated stress responses.

### 2.2. OsLAC12 Overexpression Increases Laccase Activity as Well as Lignin Biosynthesis

**Figure 2:**
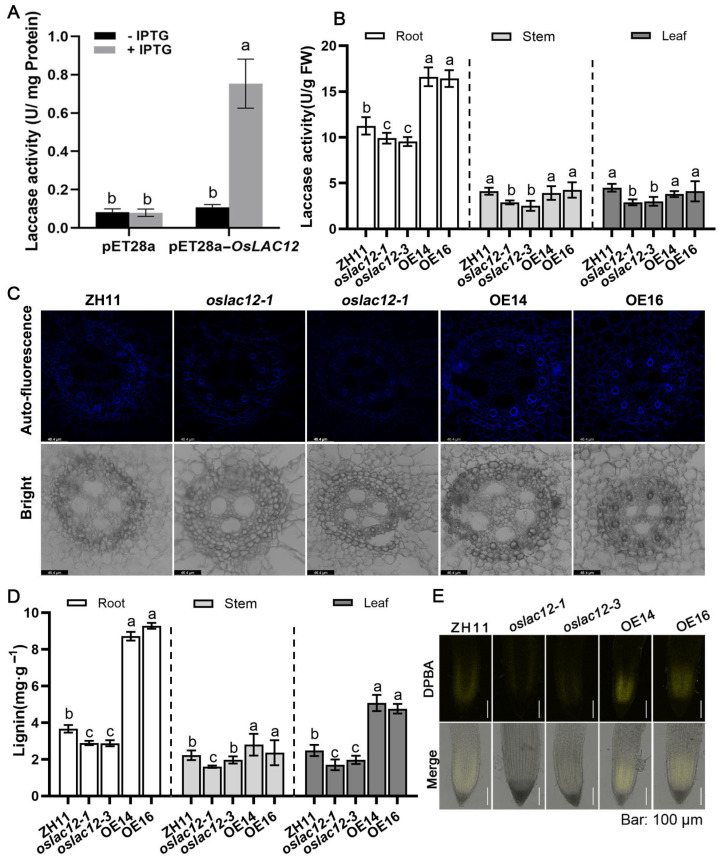
Comparative analysis of laccase activity and the content of lignin and flavonoid in ZH11, *oslac12* mutants, and overexpression lines. (**A**) Intracellular laccase activity of the *E. coli* BL21(DE3) strain expressing *OsLAC12*. (**B**) Comparative analysis of laccase activity in roots, stems, and leaves of ZH11, *oslac12* mutants, and *OsLAC12* overexpression lines, respectively. (**C**) Lignin autofluorescence in root. Scale bar = 46.4 μm. (**D**) Comparative analysis of lignin content in roots, stems, and leaves of ZH11, *oslac12* mutants, and *OsLAC12* overexpression lines, respectively. (**E**) DPBA staining showing flavonoid accumulation patterns in roots. Values represented mean ± standard deviation of three independent biological replicates. Different letters above bars denoted significant differences (*p* ≤ 0.05, ANOVA with Duncan’s test).

To verify whether the protein encoded by *OsLAC12* functions as a laccase, we constructed a recombinant plasmid *pET28a-OsLAC12* and transformed it into *E. coli* BL21 (DE3) to assess the laccase activity of the fusion protein. Following induction with isopropyl β-d-1-thiogalactopyranoside (IPTG), *E. coli* harboring the transgenic *pET28a-OsLAC12* plasmid exhibited significantly increased laccase activity, which was 9.14-fold higher than that of the empty vector control (Figure 2A). To further confirm the biological function of *OsLAC12*, we generated *OsLAC12* mutant lines and overexpression transgenic rice. Ten CRISPR/Cas9-edited transgenic lines were obtained and verified by sequencing. Two mutant alleles, designated *oslac12-1* and *oslac12-2*, were identified, carrying an insertion of a cytosine (C) or adenine (A) at position 67 of the first exon of *OsLAC12*, respectively (Figure S2A). These single-site insertion mutations resulted in the laccase, which originally encodes 682 amino acids, producing a truncated incorrect protein of 202 amino acids. An additional mutant line (03Z11CJ83) was acquired from the Rice Mutant Database (RMD). Genomic DNA amplification and analysis confirmed that this line contains a T-DNA insertion in the second exon of *OsLAC12* (Figure S2B). RT-PCR analysis revealed that this line is a *OsLAC12* loss-of-function mutant, thus it was named *oslac12-3* (Figure S2B). Transgenic rice plants overexpressing *OsLAC12* were generated using the enhanced cauliflower mosaic virus *35S* promoter. RT-PCR analysis confirmed that *OsLAC12* expression was significantly upregulated in all overexpression transgenic lines (Figure S2C).

Next, we compared the laccase activity of *oslac12-1*, *oslac12-3*, overexpression lines (OE14 and OE16), and the wild-type (ZH11) at the seedling stage. As shown in Figure 2B, laccase activity was significantly lower in the roots, stems, and leaves of the mutant lines compared to ZH11, whereas the overexpression (OE) lines exhibited higher laccase activity, with root laccase activity reaching ~1.5-fold that of the WT. To further confirm the effect of *OsLAC12* overexpression on lignin biosynthesis, total lignin contents in roots, stems, and leaves were determined using the acetyl bromide method. Consistent with the laccase activity results, *oslac12* mutants showed reduced lignin accumulation (Figure 2D). By comparison, lignin content in the roots of OE lines was significantly higher than that in the WT, reaching nearly three times the WT level; similarly, lignin content in OE stems was also significantly elevated relative to the WT, reaching twice the WT level. Additionally, observations of lignin autofluorescence in root cross-sections revealed that secondary wall thickening was correspondingly reduced in *oslac12* mutants but enhanced in *OsLAC12* OE transgenic lines (Figure 2C).

We also investigated whether *OsLAC12* overexpression alters flavonoid biosynthesis by visualizing in vivo flavonoid fluorescence. Seven-day-old seedlings were soaked in a solution containing diphenylboric acid-2-aminoethyl ester (DPBA), and flavonols were observed under a confocal microscope. Interestingly, a stronger fluorescence signal was detected in *OsLAC12* overexpression lines, whereas the mutant lines showed a slightly weaker signal (Figure 2E). Taken together, these results demonstrate that *OsLAC12* encodes a functional laccase; loss of *OsLAC12* function impairs laccase activity, while its overexpression enhances laccase activity, promotes lignin accumulation and increases flavonoid levels.

### 2.3. OsLAC12 Positively Enhances Themotolerance in Seedling Stage

**Figure 3:**
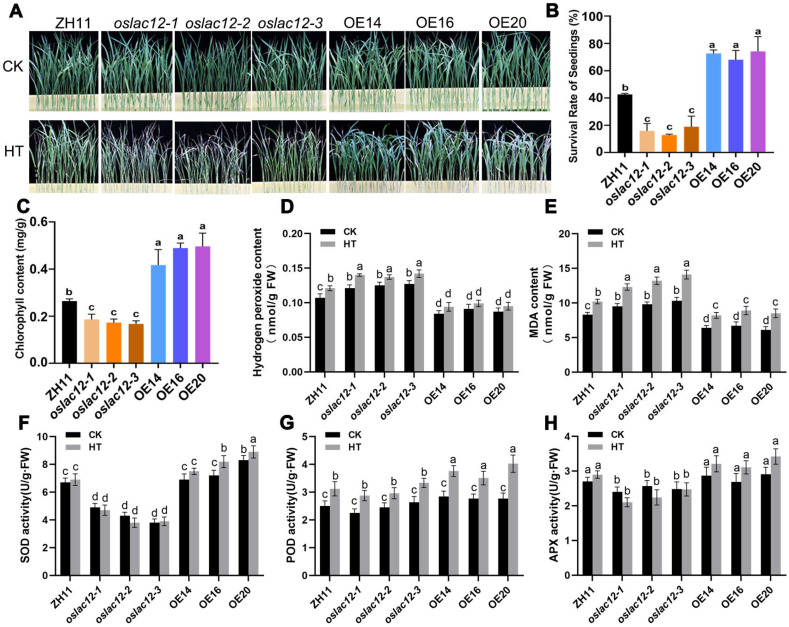
Functional characterization of *OsLAC12* in heat tolerance at seedling stage. (**A**) Phenotypic analysis of *oslac12* mutants and *OsLAC12* overexpression lines in response to HT. (**B**) Plant survival rates analysis after HT. (**C**) Leaf Chlorophyll content analysis after HT. (**D**–**H**) Analysis of antioxidant enzyme activities and ROS levels in rice seedlings response to HT. (**D**) MDA contents. (**E**) H_2_O_2_ contents. (**F**) SOD activities. (**G**) POD activities. (**H**) APX activities. Rice seedlings were exposed to 42 °C treatment for 3 days. Values represented mean ± standard deviation (*n* = 3). Different letters above bars indicated significant differences (*p* ≤ 0.05, ANOVA with Duncan’s test).

Given that *OsLAC12* expression is induced by SA and high temperature (HT), we hypothesized that *OsLAC12* contributes to heat stress responses. To test this, we compared the heat tolerance of *oslac12* mutants, overexpression lines (OE14, OE16, OE20), and wild-type ZH11 using 16-day-old hydroponically grown seedlings. Seedlings were subjected to 45 °C treatment for 30 h, followed by a 3-day recovery period at 28 °C. As shown in Figure 3A, the survival rates of *oslac12* mutants were significantly lower (~19% for *oslac12-1*, ~13% for *oslac12-2*, and ~22% for *oslac12-3*) compared to wild-type plants (~43%) (Figure 3B). In contrast, the survival rates of OE14, OE16, and OE20 were ~72.6%, 68%, and 74%, respectively, significantly higher than that of the wild type (Figure 3B). High temperature induces leaf desiccation and alters leaf color at the seedling stage; thus, we measured chlorophyll content before and after HT stress. HT-induced chlorophyll reduction was more severe in *oslac12* mutants, whereas OE14, OE16, and OE20 retained significantly higher chlorophyll content compared to ZH11 after stress (Figure 3C).

We further analyzed antioxidant enzyme activities in ZH11, *oslac12* mutants, and *OsLAC12* OE lines under both normal and HT conditions. Under normal growth conditions, SOD and APX activities were significantly lower in *oslac12* mutants than in ZH11 (Figure 3F,H), while no significant difference was observed in POD activity (Figure 3G). After HT treatment, SOD, POD, and APX activities were more strongly activated in OE lines, whereas *oslac12* mutants exhibited lower enzyme activities relative to ZH11 (Figure 3F–H). Correspondingly, *OsLAC12* OE lines maintained lower levels of malondialdehyde (MDA) and hydrogen peroxide (H_2_O_2_), while *oslac12* mutants showed the opposite trend with relatively higher accumulation of these oxidative stress markers (Figure 3D,E). Collectively, these data indicate that *OsLAC12* plays a positive role in enhancing heat tolerance by activating ROS scavenging enzymes to maintain ROS homeostasis.

### 2.4. OsLAC12 Alleviates HT-Induced ROS Brust, Pollen Viability and Spikelet Sterility

**Figure 4:**
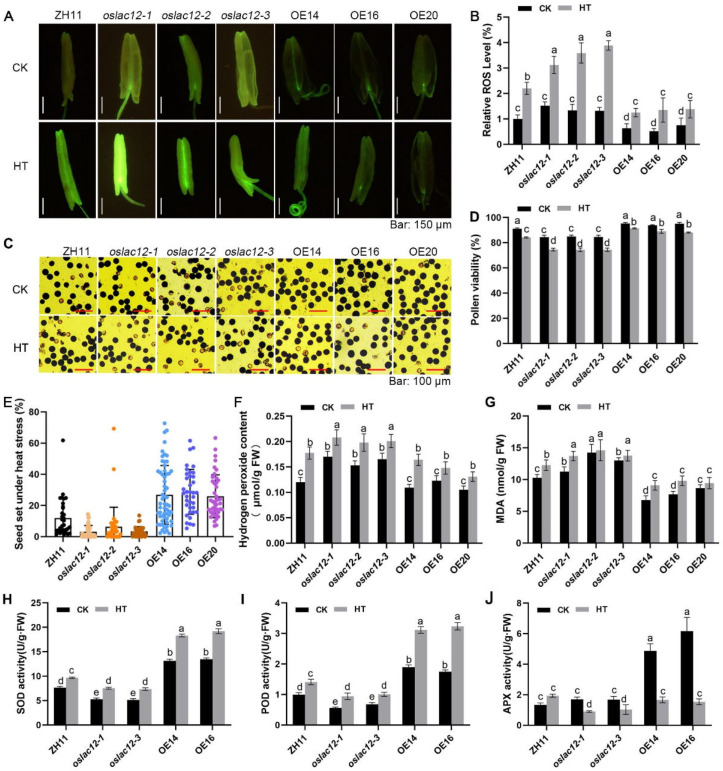
*OsLAC12* affected pollen viability and spikelet sterility under heat stress. (**A**) The H_2_DCFH-DA staining fluorescence images of the anthers under CK and HT. (**B**) Analysis of the fluorescence intensity from the fluorescence images (*n* = 10) by using ImageJ software (1.54 g). (**C**) Images of pollen grains by I_2_-KI staining in *OsLAC12* mutants and overexpression lines under CK and HT. (**D**) Analysis of the pollen viability from I_2_-KI staining images (*n* = 10). (**E**) Analysis of seed-setting rate after HT (*n* = 30). (**F**) H_2_O_2_ concentration of spilketes in response to HT. (**G**) MDA concentration of spilketes in response to HT. (**H**) SOD activities of spilketes in response to HT. (**I**) POD activities of spilketes in response to HT. (**J**) APX activities of spilketes in response to HT. Values represent mean ± SD, based on at least three independent biological replicates. Different letters above bars indicated significant differences (*p* ≤ 0.05, ANOVA with Duncan’s test).

To clarify the function of *OsLAC12* during the heading stage under HT stress, ZH11, *oslac12* mutants, and *OsLAC12* overexpression (OE) lines at the meiotic stage were exposed to normal temperature (26 °C, CK) or HT (38 °C) for 7 days, followed by recovery under normal growth conditions. We first assessed ROS accumulation in developing anthers using the fluorescent probe H_2_DCFH-DA to detect intracellular ROS. Under CK conditions, *oslac12* mutants exhibited higher anther ROS levels than ZH11, while OE lines showed lower ROS levels (Figure 4A). Upon HT treatment, HT-induced ROS bursts were significantly exacerbated in *oslac12* mutants, whereas OE lines maintained relatively lower ROS levels compared to ZH11 (Figure 4A,B). Consistent with these observations, H_2_O_2_ and MDA contents were significantly decreased in spikelets of the three OE lines but increased in those of *oslac12* mutants after HT exposure (Figure 4F,G). Further analysis of antioxidant enzyme activities in spikelets revealed that *OsLAC12* overexpression activated SOD, POD, and APX even under CK conditions (Figure 4H–J). After HT treatment, OE lines retained higher SOD, POD, and APX activities, indicating that *OsLAC12* plays a critical role in regulating ROS levels in spikelets.

Next, we analyzed pollen viability. Under CK conditions, *oslac12* mutants showed an average 10.8% reduction in pollen viability compared to ZH11 (Figure 4C,D). Under heat stress, *OsLAC12* overexpression lines maintained significantly higher pollen viability than both ZH11 and *oslac12* mutants (Figure 4C,D). As expected, seed-setting rates decreased significantly under HT, with the most severe reductions observed in *oslac12* mutants—their rates were notably lower than those of ZH11 and *OsLAC12* OE lines (Figure 4E).

### 2.5. OsLAC12 Involved in Carbohydrate Metabolism in Response to HT

**Figure 5:**
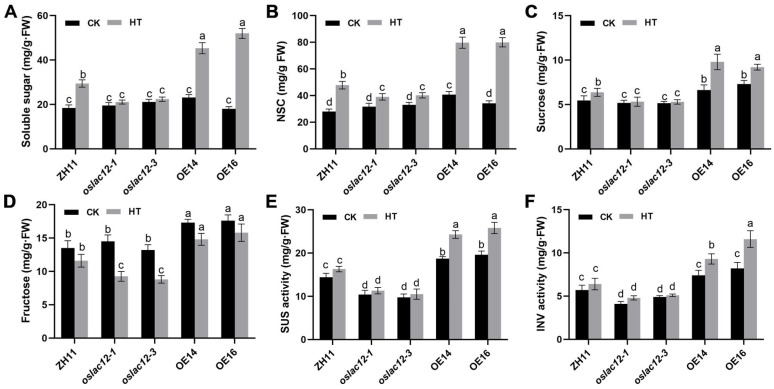
*OsLAC12* involved in regulation of sugar metabolism under heat stress. (**A**) Soluble sugar concentration of spilketes under CK and HT. (**B**) NSC conttent of spilketes under CK and HT. (**C**) Sucrose concentration of spilketes under CK and HT. (**D**) Frucrose concentration of spilketes under CK and HT. Soluble sugar concentration of spilketes under CK and HT. (**E**) SUS Activity of spilketes under CK and HT. (**F**) INV Activity of spilketes under CK and HT. Values represent mean ± SD, based on at least three independent biological replicates. Different letters above bars indicated significant differences (*p* ≤ 0.05, ANOVA with Duncan’s test).

To further investigate the impact of HT on panicle carbohydrate dynamics, we analyzed the accumulation of soluble sugars, non-structural carbohydrates (NSC), and sucrose in panicles under HT stress. As shown in Figure 5, HT induced the accumulation of these carbohydrates, and the increments were significantly more pronounced in *OsLAC12* overexpression (OE) lines than in wild-type ZH11 (Figure 5A–C). In contrast, the HT-induced increases in soluble sugars, NSC, and sucrose were markedly lower in *oslac12* mutants compared to ZH11 (Figure 5A–C). Notably, fructose content in spikelets decreased under HT, with *oslac12* mutants exhibiting the largest reduction relative to ZH11. By contrast, panicle fructose levels in *OsLAC12* OE lines remained high under both CK and HT conditions (Figure 5D). We further analyzed the activities of sucrose synthase (SUS) and acid invertase (INV)—key enzymes in sucrose metabolism that cleave sucrose into glucose and fructose—to evaluate sugar metabolism and sink strength. Under both CK and HT conditions, SUS and INV activities followed a consistent pattern: *OsLAC12* OE lines showed the highest activities, followed by ZH11, while *oslac12* mutants exhibited the lowest (Figure 5E,F). Taken together, these results indicate that *OsLAC12* acts as a positive regulator of panicle carbohydrate homeostasis under HT stress. It likely modulates spikelet development by enhancing sugar metabolism, thereby supporting sink strength and maintaining carbohydrate availability under high-temperature conditions.

## 3. Discussion

Lignin biosynthesis involves two key processes: monolignol biosynthesis and monolignol polymerization [35]. Laccases, as copper-containing oxidoreductases, catalyze the oxidation of phenolic compounds into phenoxy radicals, which subsequently undergo polymerization to form structurally complex lignin macromolecules. In this study, E. coli transformed with the *pET28a-OsLAC12* plasmid exhibited significantly higher laccase activity compared to the empty pET28a vector control (Figure 2A). More importantly, laccase activity in rice showed a close correlation with *OsLAC12* expression levels: *oslac12* mutants, which lack functional laccase activity, displayed impaired lignin accumulation in roots, stems, and leaves, whereas *OsLAC12* overexpression lines exhibited significantly elevated laccase activity accompanied by increased lignin levels (Figure 2B,C). These findings confirm that *OsLAC12* encodes a functional laccase and is involved in lignification processes.

**Figure 6:**
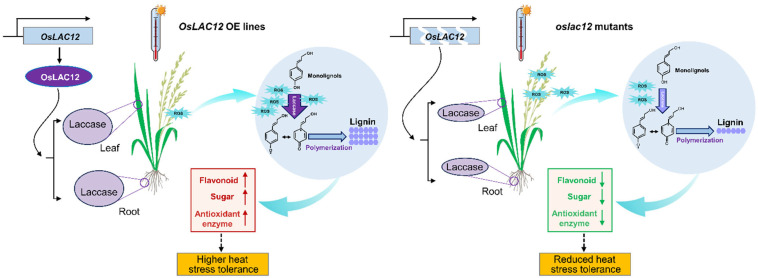
*OsLAC12* action model diagram in rice under heat stress. *OsLAC12* overexpression (OE) increased laccase activity in roots and leaves. Heat-induced ROS burst was fully used for lignin oxidation/accumulation, while elevated laccase activity may activate the phenylpropanoid pathway, promoting flavonoid synthesis, enhancing antioxidant enzyme activity mightly via phenolic metabolites, and maintaining low intracellular ROS to favor carbohydrate metabolism. These effects collectively boost plant heat tolerance. In contrast, *oslac12* mutants had low laccase activity. Heat-induced ROS utilization was limited, with reduced flavonoids and antioxidant enzymes impairing ROS scavenging. This raised intracellular ROS, disrupted carbohydrate metabolism, and ultimately decreased heat tolerance.

Laccases exhibit an extremely broad substrate range, a characteristic that contributes to their diverse and complex functions in plants, thus, many of which remain poorly characterized to date. Here, we demonstrated that *OsLAC12* plays a role in regulating heat tolerance in rice. *OsLAC12* expression is inducible by HT, SA, and ABA (Figure 1), and its involvement in heat stress responses was further validated at both the seedling and heading stages. *OsLAC12* overexpression lines, which have elevated lignin levels, maintained lower ROS accumulation and lipid oxidative damage, coupled with more robust activation of antioxidant enzyme activity, thereby sustaining reduced cellular ROS levels under HT stress (Figure 3 and Figure 4). In contrast, *oslac12* mutants displayed the opposite phenotype in response to HT (Figure 3 and Figure 4), indicating that *OsLAC12* negatively regulates HT-induced ROS accumulation. Previously, we reported that high temperature induces lignin accumulation in rice spikelets, suggesting that lignin accumulation may represent a stress-induced physiological response of rice to high-temperature stress [36]. Collectively, the present study provides evidence that this stress-induced lignification helps alleviate heat-induced damage in rice, highlighting a functional link between *OsLAC12*-mediated lignin biosynthesis and heat stress tolerance (Figure 6).

It is well established that maintaining the delicate balance between ROS-scavenging and ROS-producing systems in reproductive tissues is critical for plant responses to high temperature (HT) [9,37]. Low concentrations of ROS can activate mechanisms that mitigate oxidative stress and participate in cell wall cross-linking and lignification, thereby enhancing resistance to fungal infections [38]. However, excessive ROS accumulation, resulting from an imbalance between production and elimination, leads to irreversible oxidative damage. Plant laccases have also been implicated in ROS detoxification; unlike catalases and polyphenol oxidases, their reaction products include water, with no release of harmful ROS [39]. Notably, numerous fungal laccases can degrade toxins and a range of environmental pollutants, making laccases inherently environmentally compatible enzymes [40,41,42]. In *Camellia sinensis*, multiple laccase genes respond significantly to diverse abiotic stresses (e.g., cold, drought, high salt) and function in defense mechanisms, potentially acting as detoxifying enzymes [43,44]. In the present study, we identified that *OsLAC12* positively regulates heat tolerance by modulating ROS metabolism. Specifically, *oslac12* mutants exhibited reduced activities of SOD, POD, and APX, indicating that *OsLAC12* is associated with activating the frontline defense system responsible for ROS scavenging. Importantly, *OsLAC12* overexpression significantly enhanced the ROS scavenging system even under normal growth conditions, suggesting that this gene may constitutively activate antioxidant enzyme pathways. These findings contribute to our understanding of the detoxification function of plant laccases in coping with environmental stress.

Flavonoids, including anthocyanins, flavonols, and proanthocyanidins (PAs), protect pollen from HT stress by acting as antioxidants that scavenge ROS and control their accumulation [45,46]. In flavonoid biosynthesis, key enzymes in pathways producing major flavonoid classes, such as CHI, F3H, DFR, and ANS, have been well characterized [47,48]. Several MYB transcription factors regulate distinct branches of the phenylpropanoid metabolic pathway: for example, ectopic expression of *VvMYB5a* in tobacco promotes anthocyanin and PA biosynthesis by upregulating transcription of *CHS*, *CHI*, and *F3H*, while suppressing lignin metabolism via downregulating *CCoA-OMT1* and *CCoAOMT6* expression [49]. However, the relationship between flavonoid and lignin biosynthesis within the phenylpropanoid pathway remains poorly defined. Previous studies have shown that lignin and flavonoid yields can be modulated by regulating key genes in their biosynthetic pathways. For instance, *CsHCT*, a key lignin biosynthesis gene, increases lignin content but reduces flavonol levels when activated [50]; overexpression of *Df4CL1* and *Df4CL2* from Dryopteris fragrans promotes both flavonoid and lignin production in transgenic tobacco [51,52]; and downregulation of *GhLAC15* reduces PA and lignin contents by repressing key genes in the phenylpropanoid and flavonoid pathways [53]. In our study, differential flavonoid accumulation was observed between *OsLAC12* overexpression lines and *oslac12* mutants (Figure 2), providing evidence that altered lignin metabolism can influence flavonoid metabolism. One possible reason why *OsLAC12*-regulated changes in lignin metabolism increase flavonoid metabolism lies in the “metabolic flux expansion” of the phenylpropanoid pathway. When *OsLAC12* is overexpressed, it creates an increased demand for monolignols; this may activate upstream enzymes, which in turn expand pathway flux to boost 4-coumaroyl-CoA production, and the resulting surplus drives flavonoid synthesis. Additionally, *OsLAC12* overexpression lines showed significantly higher levels of soluble sugars and sucrose, indicating that *OsLAC12* modulates the carbohydrate composition of cell walls. Heat stress induces alterations in cell wall polymers and promotes the release of monosaccharides (e.g., xylose, arabinose) through cell wall degradation [54,55]. We therefore hypothesize that *OsLAC12* overexpression lines may enhance the metabolic conversion of these monosaccharides into soluble sugars, supplying energy to cells and thereby improving thermotolerance. Although *OsLAC12* plays important roles in regulating heat tolerance in rice, the related mechanisms remain to be clarified. In future studies, integrating transcriptomic and metabolomic analyses to identify key metabolites in the phenylpropanoid metabolic pathway, exploring the interaction between *OsLAC12* and upstream transcription factors, and investigating how this regulatory module collectively modulates rice heat tolerance will deepen our understanding of the vital role of laccases in plant responses to high-temperature stress.

## 4. Materials and Methods

### 4.1. Plant Materials

The sequence of *OsLAC12* (Gene ID: Os03g11864) was obtained from the phytozome database (https://phytozome-next.jgi.doe.gov/) (accessed on 1 August 2024). *For oslac12 mutants*, *oslac12-1* and *oslac12-2* were generated via CRISPR/Cas9 gene editing followed as guide RNA sequence used was “GCCTCTTCACTAGCGTCTCCTGG”. Meanwhile, *oslac12-3* was obtained from the Rice Mutant Database. The gene editing sites and T-DNA insertion positions were finally determined by amplifying the target fragments, conducting sequencing, and performing sequence alignment (Figure S1). Furthermore, the transcript of *oslac12-3* was analyzed via RT-PCR (Figure S1). Three *oslac12* mutants used in this study were developed with *japonica* cultivar Zhonghua11 (ZH11) as the genetic background. The coding sequence of *OsLAC12* was inserted into pGreen II [56] and introduced into *Agrobacterium tumefaciens* strains GV3101 and then transformed into ZH11 to generate transgenetic plants. For the *OsLAC12*-overexpressing transgenic rice plants, the genomic DNA tests (Figure S1) were used to select the T_1_ progenies. RT–PCR was performed to determine the transcriptional levels of *OsLAC12* in different lines, compared with WT (ZH11) plants, using the following cycling conditions: initial denaturation at 94 °C for 5 min, followed by 35 cycles of denaturation at 94 °C for 30 s, annealing at 58 °C for 30 s, and extension at 72 °C for 45 s, with a final extension at 72 °C for 10 min. Primers used are listed in Supplemental Table S1.

### 4.2. Stress Treatments and qRT-PCR Analysis

For heat stress and phytohormone treatments, seedlings of Nipponbare (*Oryza sativa* subsp. *japonica* ‘Nipponbare’) were used, following methods described previously [36,56]. For heat stress treatment, seedlings were exposed to continuous high temperature (HT; 42 °C) in a growth chamber, with sampling time points set at 0, 3, 5, 12, 24, 36, or 48 h post-treatment initiation. For phytohormone treatments, seedlings were subjected to root immersion in solutions of different hormones, including 100 μM abscisic acid (ABA), 10 mM salicylic acid (SA), 10 mM gibberellic acid (GA_3_), and 10 mM ethylene (ETH), respectively. For the combined heat and SA treatment (HT + SA), seedlings were simultaneously exposed to continuous 42 °C heat in the growth chamber and subjected to root immersion in 10 mM SA solution, with the same sampling time points as the individual heat stress treatment. Samples were collected at the indicated time points for quantitative real-time PCR (qRT-PCR) analysis.

Total RNA was extracted from seedling after various treatments using Trizol reagent (Invitrogen, Shanghai, China) according to the manufacturer’s instructions. RNA quality was assessed by 1.2% formaldehyde-denatured agarose gel electrophoresis and spectrophotometry. cDNA synthesis was performed using a PrimeScript™ RT reagent kit (Takara, Beijing, China) with a gDNA removal step. Quantitative real-time PCR (qRT-PCR) was performed using a SYBR Premix Ex Taq II kit (Takara, Wuhan, China) on a QuantStudio 6 Flex Real-Time PCR System (Thermo Fisher Scientific, Beijing, China), with the cycling parameters: 95 °C for 30 s, followed by 40 cycles of 95 °C for 5 s and 60 °C for 34 s. *Tublin* was used as an internal control for normalization as previously reported [36]. Relative gene expression levels were calculated using the 2−^ΔΔCT^ method.

### 4.3. GUS Staining

A ~1.5 kb promoter DNA fragment of *OsLAC12* upstream from start codon was constructed into pGreenII-GUS vector [56]. The *proOsLAC12::GUS* reporter construct was transformed into ZH11 using agrobacterium-mediated transformation. Transgenic plants harboring the GUS reporter gene were selected, and GUS staining was performed using the β-Glucuronidase (GUS) reporter gene kit (Thermo Fisher Scientific, Beijing, China) according to the manufacturer’s protocol. Chlorophyll was removed by incubation in 70% ethanol. GUS-stained tissues were observed and photographed using microscope (Leica TCSSP8, Wetzlar, Germany).

### 4.4. Heat Stress Application at Seedling Stage

Seeds of mutants, wild-type plants, and T_3_-generation homozygous overexpression (OE) lines were first soaked in prochloraz for 2 h, followed by thorough rinsing with distilled water and overnight soaking. On the following day, the seeds were placed in a moist environment at 30 °C to promote germination. Germinated seeds were then transferred to bottomless 96-well plates and hydroponically cultured in 1× nutrient solution within a light incubator set at 26 °C, under a 16:8 light: dark photoperiod.

Once the seedlings reached the two-leaf-one-heart stage, they were exposed to high-temperature stress at 45 °C in growth chamber until obvious wilting phenotypes became apparent. Afterward, the seedlings were allowed to recover under normal growth conditions at 26 °C for 3 days.

Subsequently, seedling survival rates were calculated, and chlorophyll content was measured and analyzed. The chlorophyll content was measured by assay kits from Solarbio (Beijing, China). Rice leaves were harvested before and after heat stress, then measure antioxidant enzymes, H_2_O_2_, and MDA.

### 4.5. Determination of SOD, POD, APX, H_2_O_2_, and MDA

The enzyme activity of *SOD*, *POD*, *APX* and MDA content were determined according to a previously described method [36,57]. Frozen seedlings (0.5 g) were ground to a fine powder in liquid nitrogen and homogenized in 50 mM sodium phosphate buffer (pH 7.0). The homogenate was centrifuged at 13,000× *g* for 15 min at 4 °C, and the resulting supernatant was aliquoted and used for subsequent analyses. Hydrogen peroxide content was determined using a hydrogen peroxide assay kit (BC3590, Solarbio). To ensure reliability, each assay was repeated with at least three biological replicates.

### 4.6. Heat Treatment on the Reproductive Stage

Rice plants were grown until the meiotic stage, identified by approximately 2 cm separation between the pulvini of the first and second uppermost leaves. Following this, plants were transferred to a growth chamber (AGC-MR, Zhejiang Qiushi Environment Co., Ltd., Zhejiang, Hangzhou, China) and subjected to a heat treatment regime mimicking local heat stress patterns. This regime consisted of a 14 h day/10-h night cycle with gradually increasing temperatures. Daily temperatures ranged from 26 °C to 38 °C, with a daily maximum temperature of 38 °C. After three days of treatment, spikelets were harvested from ZH11, mutant, and overexpression lines for antioxidant enzyme activity assays and ROS detection. Pollen viability and pollination properties were evaluated during anthesis stage.

### 4.7. Detection of ROS via DCFH-DA

To determine the levels of ROS within cells of targeted samples, a fluorescent probe, H_2_DCFDA (Sigma, Shanghai, China), was employed, as previously described by Zhao et al. [58]. Unopened spikelets of rice at the flowering stage were collected around 9:00 a.m. Anthers were dissected out and soaked in 1× PBS, followed by incubation at 37 °C for 30 min. Subsequently, the solution was replaced with 1× PBS containing 10 μM H_2_DCFDA for staining over 1 h. After staining, the anthers were rinsed at least five times with PBS to remove residual H_2_DCFDA. Fluorescence was observed and captured using a laser confocal microscope (Leica TCSSP8, Wetzlar, Germany), with excitation and emission wavelengths of 488 nm and 504–529 nm, respectively. ImageJ software was employed to determine the relative fluorescence intensity (IOD/area) of the images, with all photos analyzed under identical parameter settings.

### 4.8. Determination of Pollen Viability and Seed-Setting Rate

Pollen viability under heat stress and control conditions was assessed using a standard iodine-potassium iodide (I_2_-KI) staining method. Released pollen was stained with a 1% solution on glass slides and observed using a Leica DM4000B light microscope (Wetzlar, Germany) at 100× magnification. Pollen grains were classified as fertile or sterile based on staining characteristics. A minimum of 500 grains were evaluated per image across ten biological replicates. Pollen viability was calculated as the percentage of fertile grains.

At maturity, rice plants were harvested to count the number of filled grains and abortive grains per panicle. The seed-setting rate was calculated as (number of filled grains/total number of grains) × 100%.

### 4.9. Determination of Laccase Activity Assay

Laccase activity was determined following the method of Ding et al. with slight modifications [25]. Briefly, 0.1 g rice tissue was ground to a fine powder in liquid nitrogen, then mixed with plant protein extraction buffer (1:99, containing protease inhibitor cocktail) and vigorously shaken for 10 min. After standing on ice for 20 min, the mixture was centrifuged at 12,000× *g* for 20 min at 4 °C, and the supernatant was collected for use. The substrate ABTS was dissolved in DMSO to a final concentration of 11 g/L and stored at −20 °C. The reaction system (100 μL total) contained 100 mmol/L acetate buffer (pH 5.0), 1 mmol/L ABTS, and 20 μL protein extract. After incubation at 30 °C for 0.5 h, absorbance was measured at 420 nm, with ABTS oxidation stability used to evaluate laccase activity.

### 4.10. Determination of Lignin Content

Lignin content was measured by the thioglycolic acid method with minor modifications [36]. Briefly, 0.1 g rice powder was treated with pre-cooled 80% methanol (4 °C), shaken (250 rpm, 10 min) and centrifuged (12,000× *g*, 10 min, 4 °C). The pellet was dried (65 °C), incubated with 2 M HCl and thioglycolic acid (95 °C, 4 h), cooled on ice, then centrifuged. The pellet was washed, resuspended in 0.5 M NaOH, shaken (250 rpm, 18 h, 25 °C) and centrifuged. The pellet was re-extracted with 0.5 M NaOH; combined supernatants were mixed with concentrated HCl and kept at 4 °C for 4 h. The precipitate was dissolved in 0.5 M NaOH, and absorbance at 280 nm was measured. Content was calculated using a lignin standard curve (0–0.5 mg/mL, AccuStandard, CAS#9005-53-2) with equation y = 5.39x − 0.0298 (R^2^ = 0.999).

### 4.11. Lignin Autofluorescence Analysis

Four-week-old rice roots (young tissues) were fixed in FAA fixative (formaldehyde:glacial acetic acid:50% alcohol  =  1:1:17) for 48 h. Fixed tissues are gradually dehydrated using a series of ethanol (EtOH) solutions to remove water. Then dehydrated tissues are treated with a clearing agent to remove ethanol and make the tissue permeable to paraffin. After paraffin infiltration and embedding, sections with a thickness of 10 μm were produced using an RM2235 microtome (Leica, Wetzlar, Germany). Then, these sections were used to observe lignin autofluorescence under 405 nm UV light using a fluorescence microscope (Leica TCS SP5).

### 4.12. DPBA Staining

To assess flavonol accumulation within the roots, in situ staining with DPBA was performed. The root of 10-days-seedlings were stained with a solution containing 0.25% (*w*/*v*) DPBA and 0.01% (*v*/*v*) Triton X-100 in ethanol until a saturated signal was observed (at least 1.5 h). The resulting fluorescence was examined using a laser confocal microscope (Leica, Wetzlar, Germany) with an excitation wavelength of 458 nm.

### 4.13. Determination of Carbohydrate Content, SUS and INV Activity

The content of various carbohydrates was determined according to a previously described method [59]. About 0.5 g of frozen spilkelets powder was extracted with deionized wate. For ssoluble sugar content determination, 250 µL extract mixed with anthrone ethyl acetate reagent and concentrated sulfuric, and heated in boiling water, then measured absorbance at 630 nm against blank. For sucrose content determination, extracted supernatant was mixed with 2 M NaOH, heated in boiling water to remove impurities, then mixed with 0.1% (*m*/*v*) resorcinol in 95% ethanol and 10 M HCl. The mixture was incubated in an 80 °C water bath for 1 h, and absorbance was measured at 500 nm. For fructose content, 1 mL of supernatant was mixed sequentially with 0.1% resorcinol and 10 M HCl, incubated in an 80 °C water bath for 30 min, and absorbance was recorded at 480 nm.

For starch content determination, ~0.5 g of frozen spilkelets powder was mixed with 80% ethanol, incubated at 80 °C for 30 min to remove soluble sugars. The centrifuged pellet was boiled with distilled water for 15 min, then mixed with 9.2 M perchloric acid to extract starch. After centrifugation, supernatant was combined with distilled water and anthrone reagent. Absorbance was measured at 620 nm, and starch content was calculated using a glucose standard curve. Total non-structural carbohydrate (NSC) content was considered the sum of the soluble sugar and starch contents.

### 4.14. Determination of SUS and INV Activity

SUS and INV activity were extracted following the manufacturer’s instructions (Cominbio Co. Ltd., Suzhou, China) [60]. Briefly, about 0.1 g of spikelets was ground into powder and homogenized with 1 mL extraction buffer (50 mM HEPES, pH 7.5; 5 mM MgCl_2_; 1 mM EDTA-Na_2_; 0.5 mM dithiothreitol; 1% Triton X-100; 2% polyvinyl pyrrolidone; 10% glycerol). The suspension was centrifuged at 8000× *g* for 10 min at 4 °C, and the supernatant was used for enzyme activity assays. After reaction steps, absorbance was measured at 510 nm (SUS) and 540 nm (INV) to calculate enzyme activities.

## 5. Conclusions

In summary, we identified *OsLAC12* as a functional laccase gene that positively regulates heat tolerance in rice. This gene enhances thermotolerance by promoting the accumulation of flavonoids and lignin, activating the antioxidant defense and secondary metabolism, and maintaining cellular ROS homeostasis. Our findings broaden the current understanding of the role of laccases in plant responses to HT stress, highlighting *OsLAC12* as a promising target for improving rice heat tolerance through molecular breeding strategies.

## Data Availability

All relevant data can be found and available within the manuscript.

## Supplementary Materials

The following supporting information can be downloaded at: https://www.mdpi.com/article/10.3390/plants14182846/s1, Figure S1. Expression patterns of *OsLAC12* when *18S rRNA* as an internal control. Figure S2: Identification of *oslac12* mutants and *OsLAC12* OE lines; Table S1: PCR primer sequences used for this research.

## References

[1] Xu Y., Chu C., Yao S. (2021). The impact of high-temperature stress on rice: Challenges and solutions. Crop J..

[2] Xu J., Henry A., Sreenivasulu N. (2020). Rice yield formation under high day and night temperatures-a prerequisite to ensure future food security. Plant Cell Environ..

[3] Peng S., Huang J., Sheehy J.E., Laza R.C., Visperas R.M., Zhong X., Centeno G.S., Khush G.S., Cassman K.G. (2004). Rice yields decline with higher night temperature from global warming. Proc. Natl. Acad. Sci. USA.

[4] Prasad P.V.V., Boote K.J., Allen L.H., Sheehy J.E., Thomas J.M.G. (2006). Species, ecotype and cultivar differences in spikelet fertility and harvest index of rice in response to high temperature stress. Field Crops Res..

[5] Endo M., Tsuchiya T., Hamada K., Kawamura S., Yano K., Ohshima M., Higashitani A., Watanabe M., Kawagishi-Kobayashi M. (2009). High temperatures cause male sterility in rice plants with transcriptional alterations during pollen development. Plant Cell Physiol..

[6] Altenbach S.B., DuPont F.M., Kothari K.M., Chan R., Johnson E.L., Lieu D. (2003). Temperature, water and fertilizer influence the timing of key events during grain development in a US spring wheat. J. Cereal Sci..

[7] Jagadish S.V., Muthurajan R., Oane R., Wheeler T.R., Heuer S., Bennett J., Craufurd P.Q. (2010). Physiological and proteomic approaches to address heat tolerance during anthesis in rice (*Oryza sativa* L.). J. Exp. Bot..

[8] Zhang G., Zhang S., Xiao L., WU X., Xiao Y., Chen L. (2013). Effect of high temperature stress on physiological characteristics of anther and pollen traits of rice at flowering stage. Acta Agron. Sin..

[9] Mittler R. (2017). ROS are good. Trends Plant Sci..

[10] Fortunato S., Lasorella C., Dipierro N., Vita F., de Pinto M.C. (2023). Redox signaling in plant heat stress response. Antioxidants.

[11] Choudhury F.K., Rivero R.M., Blumwald E., Mittler R. (2017). Reactive oxygen species, abiotic stress and stress combination. Plant J..

[12] Zhao Q., Zhou L., Liu J., Cao Z., Du X., Huang F., Pan G., Cheng F. (2018). Involvement of CAT in the detoxification of HT-induced ROS burst in rice anther and its relation to pollen fertility. Plant Cell Rep..

[13] Dumanovic J., Nepovimova E., Natic M., Kuca K., Jacevic V. (2021). The significance of reactive oxygen species and antioxidant defense system in plants: A concise overview. Front. Plant Sci..

[14] Das K., Roychoudhury A. (2014). Reactive oxygen species (ROS) and response of antioxidants as ROS-scavengers during environmental stress in plants. Front. Environ. Sci..

[15] Lu C., Li W., Feng X., Chen J., Hu S., Tan Y., Wu L. (2025). The dynamic remodeling of plant cell wall in response to heat stress. Genes.

[16] Li Z., Li Z., Ji Y., Wang C., Wang S., Shi Y., Le J., Zhang M. (2024). The heat shock factor 20-HSF4-cellulose synthase A2 module regulates heat stress tolerance in maize. Plant Cell.

[17] Boerjan W., Ralph J., Baucher M. (2003). Lignin biosynthesis. Annu. Rev. Plant Biol..

[18] Mot A.C., Silaghi-Dumitrescu R. (2012). Laccases: Complex architectures for one-electron oxidations. Biochemistry.

[19] Reiss R., Ihssen J., Richter M., Eichhorn E., Schilling B., Thöny-Meyer L. (2013). Laccase versus laccase-like multi-copper oxidase: A comparative study of similar enzymes with diverse substrate spectra. PLoS ONE.

[20] Wilson M.T., Torres J. (2004). Reactions of nitric oxide with copper containing oxidases; cytochrome c oxidase and laccase. IUBMB Life.

[21] Cai X., Davis E.J., Ballif J., Liang M., Bushman E., Haroldsen V., Torabinejad J., Wu Y. (2006). Mutant identification and characterization of the laccase gene family in *Arabidopsis*. J. Exp. Bot..

[22] Liu Q., Luo L., Wang X., Shen Z., Zheng L. (2017). Comprehensive analysis of rice laccase gene (*OsLAC*) family and ectopic expression of *OsLAC10* enhances tolerance to copper stress in *Arabidopsis*. Int. J. Mol. Sci..

[23] Wang J., Feng J., Jia W., Fan P., Bao H., Li S., Li Y. (2017). Genome-wide identification of *Sorghum bicolor* laccases reveals potential targets for lignin modification. Front. Plant Sci..

[24] Sun Z., Zhou Y., Hu Y., Jiang N., Hu S., Li L., Li T. (2022). Identification of wheat *LACCASEs* in response to *Fusarium graminearum* as potential deoxynivalenol trappers. Front. Plant Sci..

[25] Berthet S., Demont-Caulet N., Pollet B., Bidzinski P., Cézard L., Le Bris P., Borrega N., Hervé J., Blondet E., Balzergue S. (2011). Disruption of *LACCASE4* and *17* results in tissue-specific alterations to lignification of *Arabidopsis thaliana* stems. Plant Cell.

[26] Zhao Q., Nakashima J., Chen F., Yin Y., Fu C., Yun J., Shao H., Wang X., Wang Z.Y., Dixon R.A. (2013). Laccase is necessary and nonredundant with peroxidase for lignin polymerization during vascular development in *Arabidopsis*. Plant Cell.

[27] Wang X., Zhuo C., Xiao X., Wang X., Docampo-Palacios M., Chen F., Dixon R.A. (2020). Substrate specificity of LACCASE8 facilitates polymerization of caffeyl alcohol for C-lignin biosynthesis in the seed coat of *Cleome hassleriana*. Plant Cell.

[28] Zhuo C., Wang X., Docampo-Palacios M., Sanders B.C., Engle N.L., Tschaplinski T.J., Hendry J.I., Maranas C.D., Chen F., Dixon R.A. (2022). Developmental changes in lignin composition are driven by both monolignol supply and laccase specificity. Sci. Adv..

[29] Yu Y., Li Q.F., Zhang J.P., Zhang F., Zhou Y.F., Feng Y.Z., Chen Y.Q., Zhang Y.C. (2017). *Laccase-13* regulates seed setting rate by affecting hydrogen peroxide dynamics and mitochondrial integrity in rice. Front. Plant Sci..

[30] Xiao S., Ming Y., Zhou S., Dong X., Liu S., Zhang X., Zhang X., Hu Q., Zhu L. (2024). A *GhLac1*-centered transcriptional regulatory cascade mediates cotton resistance to *Verticillium dahliae* through the lignin biosynthesis pathway. Int. J. Biol. Macromol..

[31] Sun Q., Liu X., Yang J., Liu W., Du Q., Wang H., Fu C., Li W.X. (2018). MicroRNA528 affects lodging resistance of maize by regulating lignin biosynthesis under nitrogen-luxury conditions. Mol. Plant.

[32] Cho H.Y., Lee C., Hwang S.G., Park Y.C., Lim H.L., Jang C.S. (2014). Overexpression of the *OsChI1* gene, encoding a putative laccase precursor, increases tolerance to drought and salinity stress in transgenic *Arabidopsis*. Gene.

[33] Xu X., Zhang Y., Liang M., Kong W., Liu J. (2022). The citrus laccase gene *CsLAC18* contributes to cold tolerance. Int. J. Mol. Sci..

[34] Xu X., Zhang Y., Wang B., Ding L., Wang Y., Luo L., Zhang Y., Kong W. (2019). Genome-wide identification and characterization of laccase gene family in *Citrus sinensis*. Gene.

[35] Rajesh Banu J., Kavitha S., Yukesh Kannah R., Poornima Devi T., Gunasekaran M., Kim S.H., Kumar G. (2019). A review on biopolymer production via lignin valorization. Bioresour. Technol..

[36] Cai Z., He F., Feng X., Liang T., Wang H., Ding S., Tian X. (2020). Transcriptomic analysis reveals important roles of lignin and flavonoid biosynthetic pathways in rice thermotolerance during reproductive stage. Front. Genet..

[37] Suzuki N., Katano K. (2018). Coordination between ROS regulatory systems and other pathways under heat stress and pathogen attack. Front. Plant Sci..

[38] Shi L., Gong L., Zhang X.Y., Ren A., Gao T., Zhao M.W. (2015). The regulation of methyl jasmonate on hyphal branching and GA biosynthesis in *Ganoderma lucidum* partly via ROS generated by NADPH oxidase. Fungal Genet. Biol..

[39] Janusz G., Pawlik A., Świderska-Burek U., Polak J., Sulej J., Jarosz-Wilkołazka A., Paszczyński A. (2020). Laccase properties, physiological functions, and evolution. Int. J. Mol. Sci..

[40] Wang G.D., Li Q.J., Luo B., Chen X.Y. (2004). *Ex planta* phytoremediation of trichlorophenol and phenolic allelochemicals via an engineered secretory laccase. Nat. Biotechnol..

[41] Mayer A.M., Staples R.C. (2002). Laccase: New functions for an old enzyme. Phytochemistry.

[42] Zhong Z., Li N., He B., Igarashi Y., Luo F. (2019). Transcriptome analysis of differential gene expression in *Dichomitus squalens* during interspecific mycelial interactions and the potential link with laccase induction. J. Microbiol..

[43] Yu Y., Xing Y., Liu F., Zhang X., Li X., Zhang J., Sun X. (2021). The laccase gene family mediate multi-perspective trade-offs during tea plant (*Camellia sinensis*) development and defense processes. Int. J. Mol. Sci..

[44] Zhu J., Zhang H., Huang K., Guo R., Zhao J., Xie H., Zhu J., Gu H., Chen H., Li G. (2023). Comprehensive analysis of the laccase gene family in tea plant highlights its roles in development and stress responses. BMC Plant Biol..

[45] Dias M.C., Pinto D.C.G.A., Silva A.M.S. (2021). Plant flavonoids: Chemical characteristics and biological activity. Molecules.

[46] Kamble N.U. (2024). Decoding the role of flavonoids in ROS management during heat stress in tomato pollen. Plant Cell.

[47] Shen N., Wang T., Gan Q., Liu S., Wang L., Jin B. (2022). Plant flavonoids: Classification, distribution, biosynthesis, and antioxidant activity. Food Chem..

[48] Liu W., Feng Y., Yu S., Fan Z., Li X., Li J., Yin H. (2021). The flavonoid biosynthesis network in plants. Int. J. Mol. Sci..

[49] Deluc L., Barrieu F., Marchive C., Lauvergeat V., Decendit A., Richard T., Carde J.P., Mérillon J.M., Hamdi S. (2006). Characterization of a grapevine R2R3-MYB transcription factor that regulates the phenylpropanoid pathway. Plant Physiol..

[50] Chen Y., Yi N., Yao S.B., Zhuang J., Fu Z., Ma J., Yin S., Jiang X., Liu Y., Gao L. (2021). *CsHCT*-mediated lignin synthesis pathway involved in the response of tea plants to biotic and abiotic stresses. J. Agric. Food Chem..

[51] Li S.S., Chang Y., Li B., Shao S.L., Zhen-Zhu Z. (2020). Functional analysis of 4-coumarate: CoA ligase from *Dryopteris fragrans* in transgenic tobacco enhances lignin and flavonoids. Genet. Mol. Biol..

[52] Li S., Chang Y., Lin Teoh P., Wang D., Mo J., Li B., Shao S. (2021). Overexpression of *Df4CL1* from *Dryopteris fragrans* enhances flavonoids and lignin production in transgenic Tobacco. Russ. J. Plant Physiol..

[53] Jiao J., Zheng H., Zhou X., Huang Y., Niu Q., Ke L., Tang S., Liu H., Sun Y. (2024). The functions of laccase gene *GhLAC15* in fiber colouration and development in brown-colored cotton. Physiol. Plant.

[54] Lima R.B., dos Santos T.B., Vieira L.G.E., Ferrarese M.L.L., Ferrarese-Filho O., Donatti L., Boeger M.R.T., Petkowicz C.L.d.O. (2013). Heat stress causes alterations in the cell-wall polymers and anatomy of coffee leaves (*Coffea arabica* L.). Carbohydr. Polym..

[55] Barnes W.J., Anderson C.T. (2018). Release, Recycle, Rebuild: Cell-wall remodeling, autodegradation, and sugar salvage for new wall biosynthesis during plant development. Mol. Plant.

[56] Ding S., Zhang B., Qin F. (2015). *Arabidopsis* RZFP34/CHYR1, a ubiquitin E3 ligase, regulates stomatal movement and drought tolerance via SnRK2.6-mediated phosphorylation. Plant Cell.

[57] Giannopolitis C.N., Ries S.K. (1977). Superoxide dismutases: I. Occurrence in higher plants. Plant Physiol..

[58] Zhao Q., Guan X., Zhou L., Asad M.A., Xu Y., Pan G., Cheng F. (2023). ABA-triggered ROS burst in rice developing anthers is critical for tapetal programmed cell death induction and heat stress-induced pollen abortion. Plant Cell Environ..

[59] Islam M.R., Feng B., Chen T., Fu W., Zhang C., Tao L., Fu G. (2018). Abscisic acid prevents pollen abortion under high-temperature stress by mediating sugar metabolism in rice spikelets. Physiol. Plantarum..

[60] Jiang N., Yu P., Fu W., Li G., Feng B., Chen T., Li H., Tao L., Fu G. (2020). Acid invertase confers heat tolerance in rice plants by maintaining energy homoeostasis of spikelets. Plant Cell Environ..

