# Supplementary material for: OsLAC12, a Laccase-Encoding Gene, Contributes to Rice Heat Tolerance Through Regulation of Antioxidant Defense and Secondary Metabolism
Source: Plants (Basel). 2025 Sep 12;14(18):2846. doi: 10.3390/plants14182846 (PMC12473251; doi:10.3390/plants14182846)
Supplement: Supplementary file 1 [file plants-14-02846-s001.zip › Supplemental Figure.pdf]

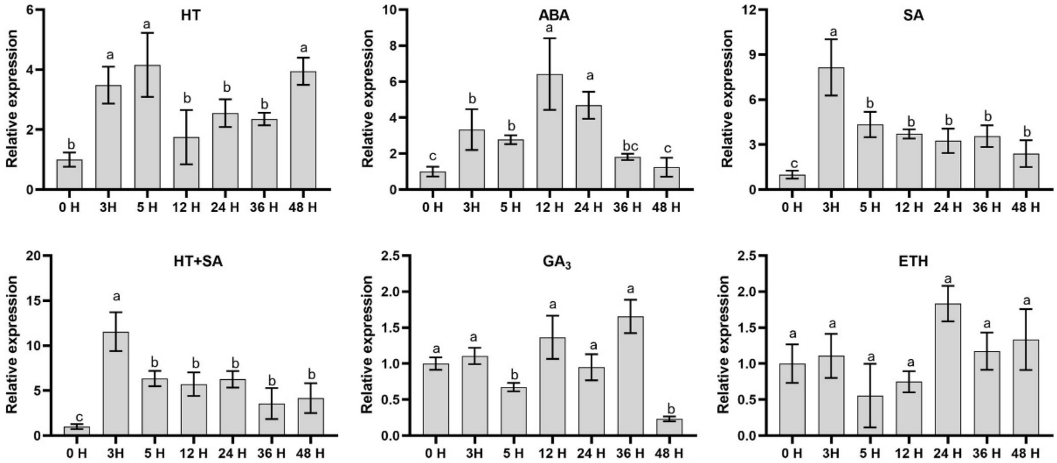

Figure S1. Expression patterns of *OsLAC12* when *18S rRNA* as an internal control. Data were presented as mean ± SD, based on three independent biological replicates. Bars with different letters indicate statistically significant differences ($P \leqq 0.05$, ANOVA with Duncan's test).

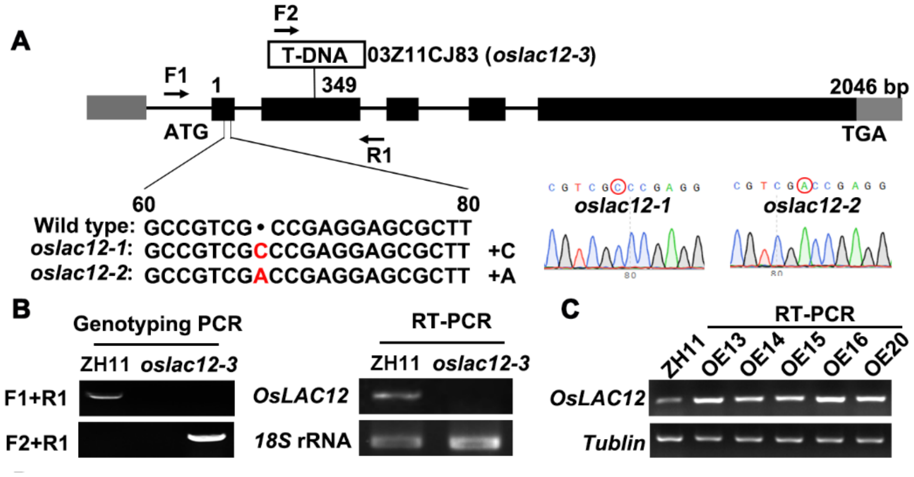

Figure S2 Identification of *oslac12* mutants and *OsLAC12* OE lines (A) Schematic representation of the *OsLAC12* gene structure. Exons: black boxes; UTRs: gray boxes; introns: lines. It was illustrated the mutation types and sites for three mutants. **(B)** PCR confirmation of the T-DNA insertion in *oslac12-3* (left) and RT-PCR analysis of *OsLAC12* expression in ZH11 and *oslac12-3* (right). **(C)** RT-PCR analysis of *OsLAC12* expression in ZH11 and *OsLAC12* overexpression lines.
